# Supplementary figures and images for: Predicting Plasmodium falciparum infection status in blood using a multiplexed bead-based antigen detection assay and machine learning approaches
Source: PLoS One. 2022 Sep 29;17(9):e0275096. doi: 10.1371/journal.pone.0275096 (PMC9521833; doi:10.1371/journal.pone.0275096)

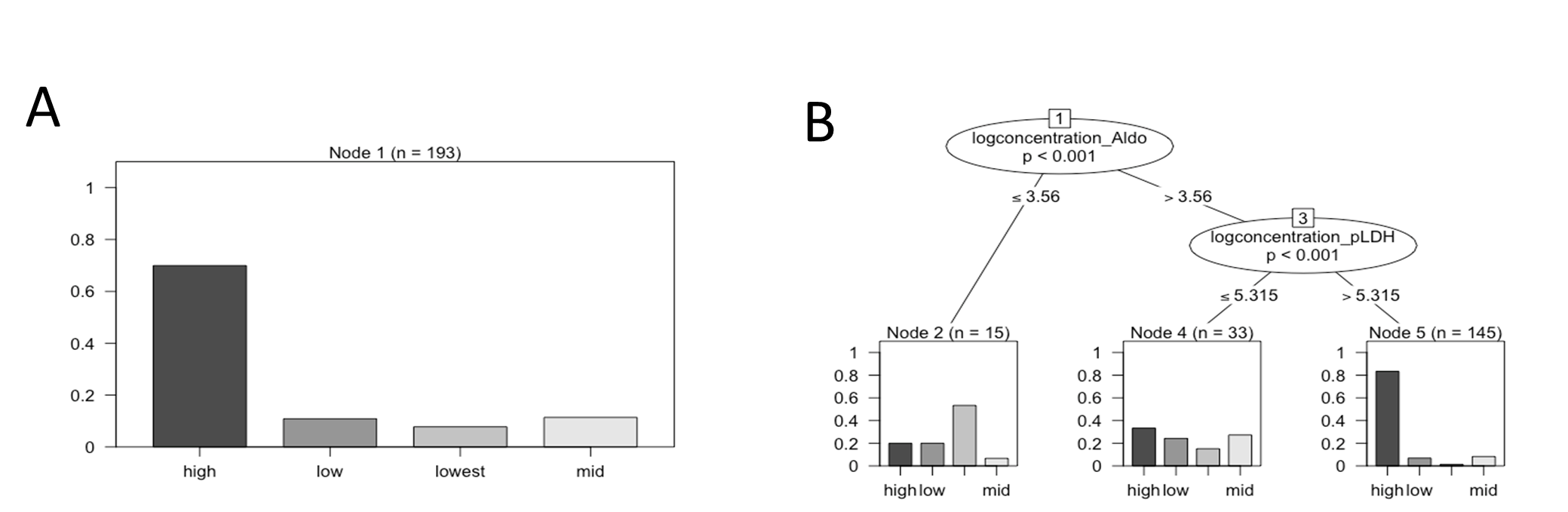

Supplement: S1 Fig — Infection level categories: Lowest = > 0–5,000; Low = > 5,000–10,000; Mid = >10,000–15,000; High = > 15,000. A) Angola (microscopy). B) Angola (microscopy), log scale. Y-axes at for all plots indicate probability of correct classification on a scale of 0.0 to 1.0. (TIF) [file pone.0275096.s001.tif]

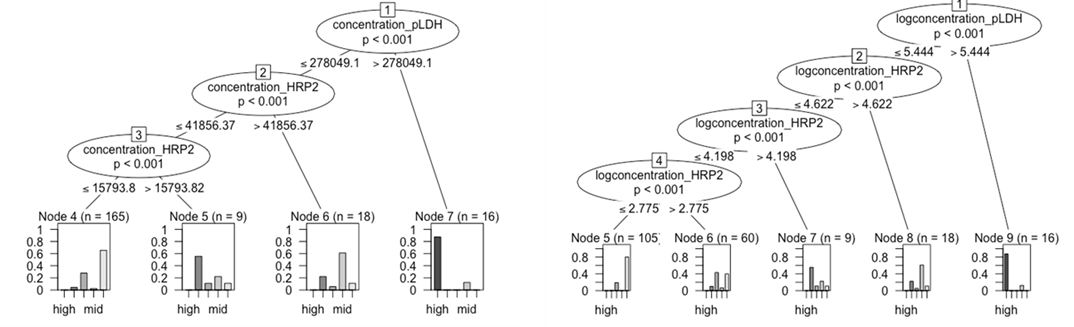

Supplement: S2 Fig — Data shown for Angola (sen-qPCR) classification with antigen concentrations on non-transformed scale on left and log-transformed on right. Y-axes at base of trees indicate probability of correct classification on a scale of 0.0 to 1.0. (TIF) [file pone.0275096.s002.tif]

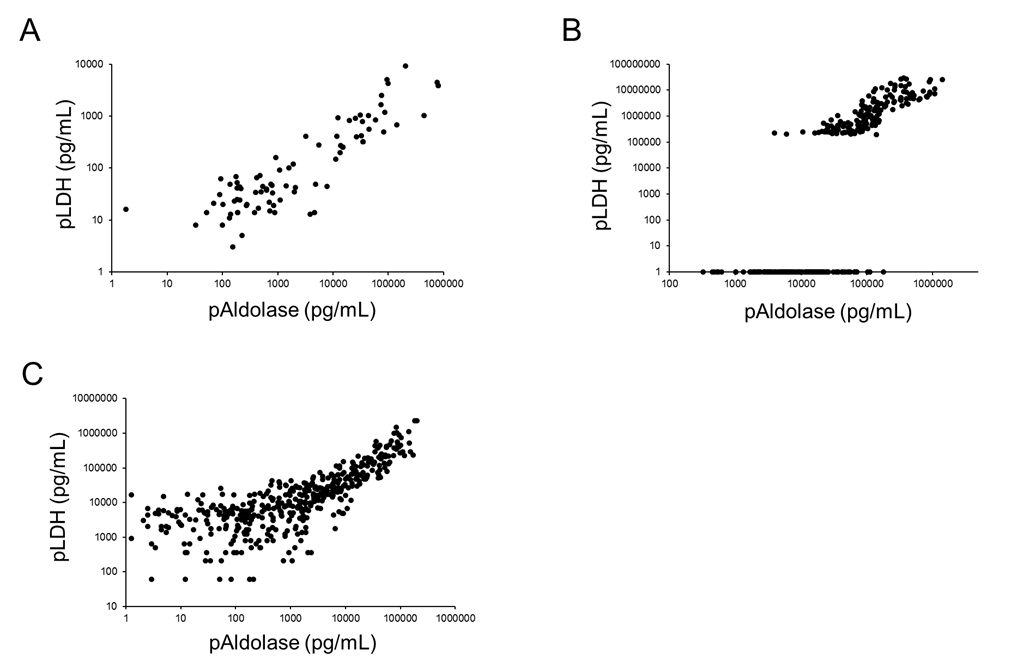

Supplement: S3 Fig — Antigen concentration data shown for the Angola 2015 TES (A), Angola 2016 health facility (B), and Haiti bednet (C) studies with concentration of pAldolase on x-axis and pLDH on y-axis for each. (TIF) [file pone.0275096.s003.tif]

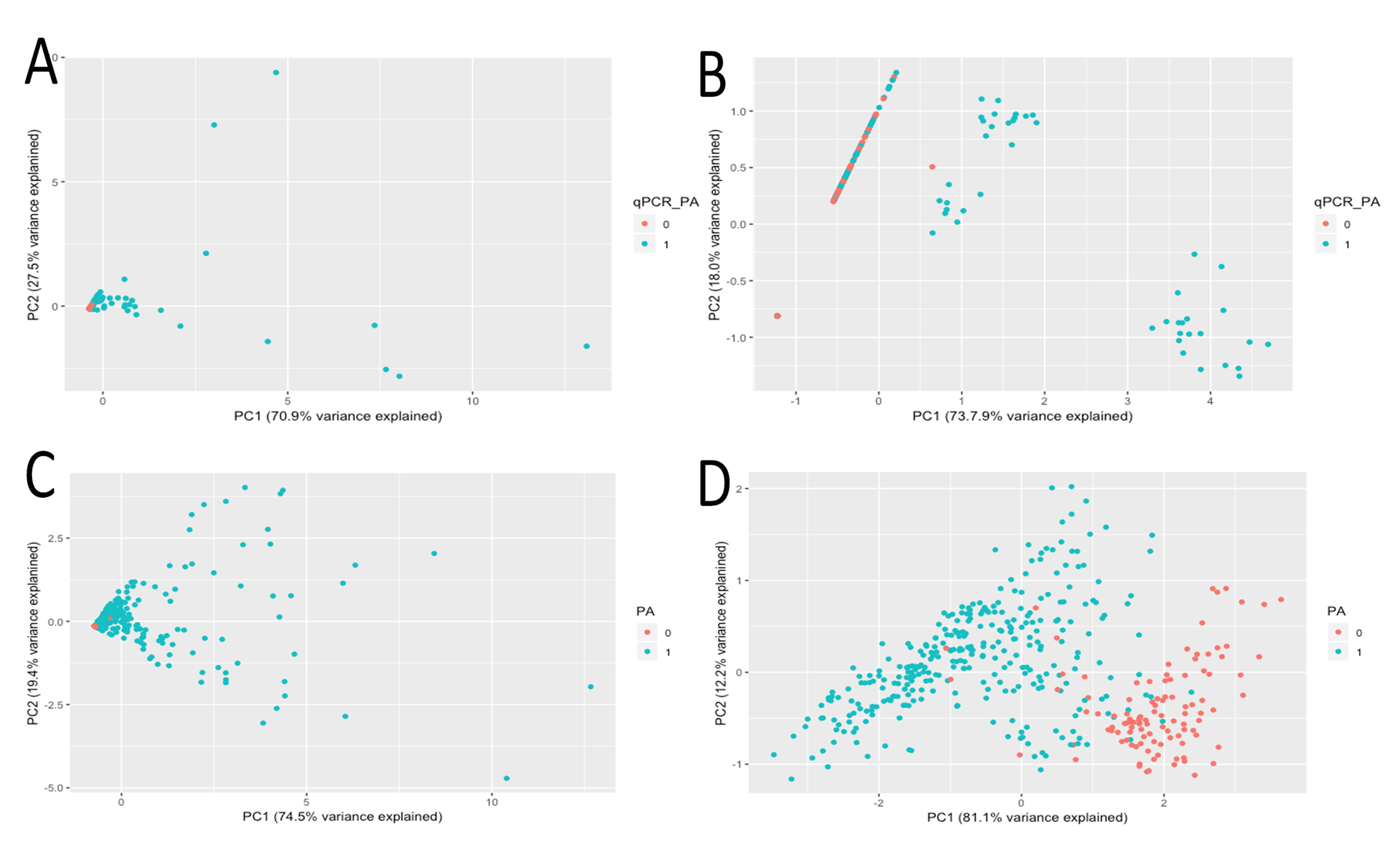

Supplement: S4 Fig — A) Angola (sen-qPCR). B) Angola (sen-qPCR), log scale. C) Haiti (PET-PCR). D) Haiti (PET), log scale. (TIF) [file pone.0275096.s004.tif]

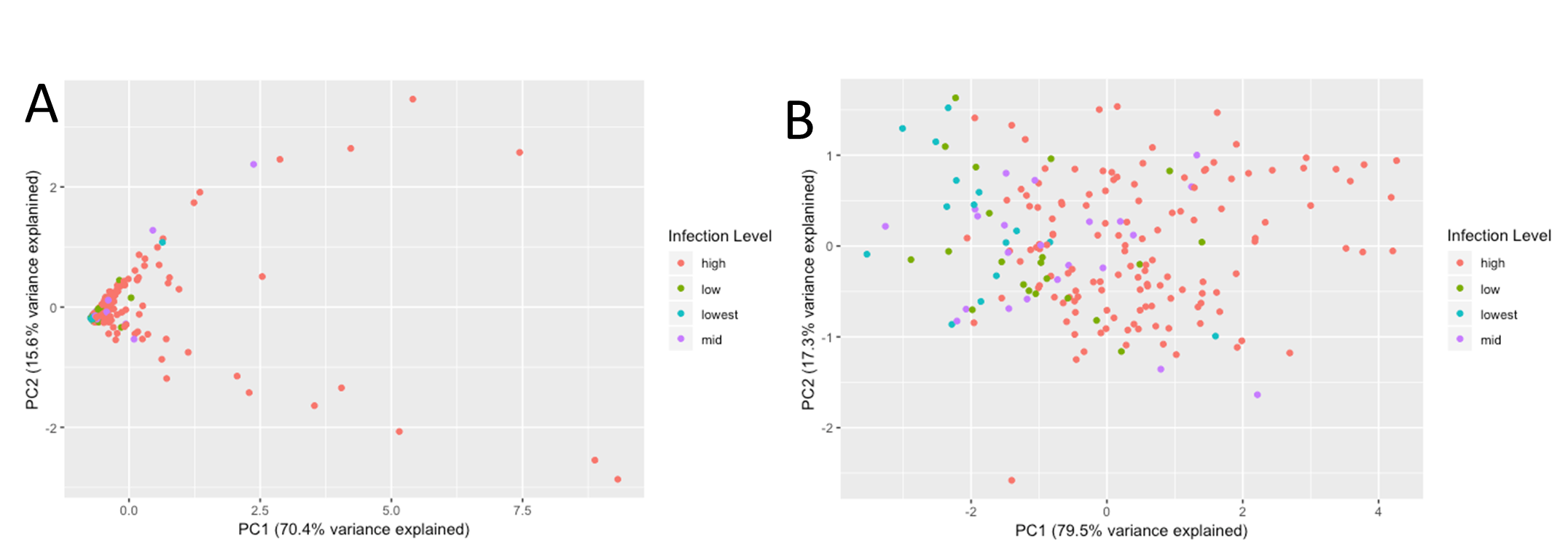

Supplement: S5 Fig — Infection level categories: Lowest = > 0–5,000; Low = > 5,000–10,000; Mid = >10,000–15,000; High = > 15,000. A) Angola (microscopy). B) Angola (microscopy), log scale. (TIF) [file pone.0275096.s005.tif]
