# Supplementary material for: Predicting Plasmodium falciparum infection status in blood using a multiplexed bead-based antigen detection assay and machine learning approaches
Source: PLoS One. 2022 Sep 29;17(9):e0275096. doi: 10.1371/journal.pone.0275096 (PMC9521833; doi:10.1371/journal.pone.0275096)
Supplement: Attachment — Submitted filename: Response to Reviewers.docx [file pone.0275096.s007.docx]

PONE-D-21-34503

Predicting Plasmodium falciparum infection status in blood using a multiplexed bead-based antigen detection assay and machine learning approaches

**Response to Editor and Reviewers**

Additional Editor Comments:

- Figures were quite blurred. Please review the PLOS One figure requirements and see whether Figures can be uploaded in a higher resolution format.
- **This has been done.**
- Do you intend to provide the code and data that underline this study?
- **Yes, we have now provided the code on Github, and indicated this at the end of Methods. We have also added a statement to the end of Methods that “Data used in these analyses is available upon request to the corresponding author”.**

**Reviewer #1:** This study examines whether the quantitative measurements of Plasmodium antigen in human exposed blood samples can be used to train machine learning algorithms to categorize patients by parasite presence or absence and parasite density level in order to achieve greater nuance in malaria diagnostics using antigen based assays rather than more costly DNA amplification methodologies. Blood samples from different cohort comprising symptomatic children and febrile or non-febrile adults were used to determine parasitemia by different quantitative methods, and antigen levels for three different antigens were determine by a bead-based quantitative assay. Conditional inference trees and PCA were performed for classification into presence or absence of parasites and into pre-defined parasite levels based on transformed and un-transformed antigen levels. Prediction of PCR infection status and parasitemia levels varied depending on antigen transformation of data.

This pilot study provides encouraging results to show that machine learning algorithms can be trained using quantitative antigen data in order to predict infection status and parasitemia levels. Results and limitations of the study and study cohort were appropriately discussed.

Minor points:
Given that the machine based learning algorithms are trained by continuous antigen data derived from dried blood spots, could the authors comment or speculate on how this would impact on the assessment of fresh blood samples in POC health care settings (i.e. would antigen measurement and DNA extraction from dried blood spots potentially underestimate parasite density due to poor antigen and DNA extraction?).

- **The reviewer brings up an important point here regarding protein and DNA integrity from different sample types. Additional text has been added to the limitations section of Discussion (Line 297) to point out that only DBS were used in these studies, and the potential for antigen/DNA degradation: “ Additionally, the only sample type utilized in these surveys was DBS, and during drying and storage, potential degradation of antigen or DNA may have occurred to understate the quantity of these biomarkers.”**

Results, lines 146ff describe the mean number of parasites/µl whereas figure 1 shows median. Could the authors make this consistent, i.e. quote median in text or show mean in figure.

- **We report the mean in the text because it is more informative than the median (for example, some of the medians are 0). Please note that the y-axis in the Figure is on a log-scale.**

Table 1: Can the authors please define sensitivity and specificity in the table notes

- **Additional text has been added to the Table 1 footnote to explain this further: “Accuracy, Se, and Sp are based on correct classification of malaria parasite presence/absence by utilizing PCR result as gold standard”.**

Font size in all figures and tables should be increased

 **- This has been done**

Figure S3: Is this a Spearman or Pearson’s correlation

- **The use of the word ‘correlation’ is not the most appropriate here, as we do not provide statistical tests to assess true correlation and significance. This word has been replaced in both the text and figure legend.**

**Reviewer #2:** The paper entitled “Predicting Plasmodium falciparum infection status in blood using a multiplexed bead-based antigen detection assay and machine learning approaches” is an application of conditional inference trees to malaria infection data. The paper is interesting and, as far as I know, it is the first time that this type of methodology was applied to malaria data; other machine learning techniques such as random forests were used in searching protective immunity against clinical malaria (doi: 10.1371/journal.pcbi.1005812) and this should be acknowledged in the Introduction.

- **We thank the reviewer for this suggestion, and this study has been acknowledged in the Introduction and is now reference #15.**

However, I think an extensive revision of the paper is needed in order to make the take-home message more compelling and crystal clear. Below please find my specific comments:

- The title does not reflect the content of the paper. First, infection levels were also predicted in the study. Second, it was only applied a single method (i.e., conditional inference trees) to predict infection status and levels and hence the use of the plural ''approaches'' is not appropriate. Third, if one tracks down the theoretical developments of conditional inference trees, they were published in statistical rather machine learning journals. Therefore, conditional inference trees are more statistical learning techniques than machine learning ones.

**-	The authors thank the reviewers for the insight. We agree with the reviewer that the main narrative of the paper is not to assess the ideal machine learning approach to this sort of dataset. Our objective was to show that a machine learning approach (like conditional inference trees) can be effective at classifying samples by infection status. There is a large overlap between “statistical approach” and “machine learning approach”, and both could apply here. From our perspective, “infection status” is inclusive of both positivity/negativity as well as infection level.**

- The motivation of this study should be rephrased more clearly. Is the motivation on the use of the methodology or the use of data from multiplex bead assays? I guess it is the methodology but then why to use conditional inference trees and exclude other existing methodologies to tackle the same classification problem?

**-	Thank you for pointing out this lack of clarity in our report. Our motivation was to apply an appropriate statistical tool (conditional inference trees) that was chosen in advance and apply it to a classification problem using data from multiplex bead assays for antigen detection. Our motivation was decidedly not to compare different methodologies and choose the most appropriate one – that would be well outside the scope of this work. However, we appreciate this comment and have added an additional paragraph near the end of Discussion explicitly stating the possibility of using alternative statistical and machine learning tools to address the same classification problem. We hope to explore other methods in future work.**

- In the introduction, it is important to be clear that absent production of the antigen target (line 71) is mostly for the HRP2 case. As far as I know, there are no reports of gene deletions for Aldolase and LDH. This might be obvious for most malaria researchers but for less specialized audience, I would write that LDH, Aldolase, and HRP2 are the proteins used in current pan-malaria and pf-malaria RDTs.

**- The authors agree with this comment and that this should be better clarified. We add additional text here to accentuate that these three antigens are the current targets for malaria RDTs, and also clarify that only HRP2 is known to have gene deletions that would affect RDT results.**

- Please provide data about the prevalence of infection for the Haiti study and the second study from Angola. It would be useful to provide information (mean, median, range, etc) about the age of the participants from each study. This increases the interpretability of the results.

- **The authors agree this is useful information for the readers’ interpretation. We have now added the requested information to the beginning of the Results section.**

- In Figure 1 (Angola sen qPCR), there are 25% of the infections that have parasitemia below 1 mu/l. Can we trust these low levels of parasite density? What is the lower level of detection above which one can trust the respective parasitemia quantification? This point is important to clarify given that the limited performance of the conditional inference trees might be caused by these infections with low parasitemia.

- **To provide the reader with additional information, we have added the analytical sensitivity of this qPCR assay to line 119 of Methods with appropriate reference: “…, with an analytical sensitivity of 0.02 parasites/µL (19).”**

- Is there any rationale to divide infection levels into the 5 categories used? If this categorization is completely arbitrary, this should be clearly stated. Otherwise, provide a rationale (maybe related to the expected sensitivity to RDT as function of parasitemia).

- **The reviewer is correct regarding the RDT test reliability assumption, and** **the categories were selected on a log10 scale with the 200p/uL as the benchmark being the minimum parasite density RDT product testing employs. The authors have added additional text to line 137 in Methods to explain this.**

- In the materials & methods, provide a brief explanation about conditional inference trees, how they are constructed and interpreted. This increases readability of the paper to a less specialized audience.

- **The authors have added an enhanced explanation of conditional inference trees in the “Data analysis and malaria infection status classification” section.**

- It is worth mentioning that conditional inference trees are dependent on the scale of covariates/features. This is a limitation of the methodology that should be acknowledged. This limitation could have been avoided by using other methodologies, such as random forest or XGBoost, which are invariant to change of scale. Why were not these methodologies applied to the same data?

- **The authors appreciate the comment and have added an additional paragraph to Discussion that proposes future alternate statistical approaches including k-nearest neighbor regression, linear discrimination analysis, random forest, gradient boosting, or finite mixture models.**

- It is also unclear whether simpler and more common approaches such as logistic regression, probit regression or other generalized models for binary/categorical data could perform equally well in the same data. Linear discrimination analysis is also another population alternative for classification problems using multivariate data.

- **The authors appreciate this comment, and will refer the reviewer to the comment above regarding other statistical approaches. The authors have updated Discussion to explicitly mention alternative statistical approaches.**

- It was used a leave-one-out cross-validation procedure. This allows the estimation of the sensitivity (Se) and specificity (Sp) shown in Table 1. But I think 5-fold or 10-fold cross-validation provides a better idea of how robust (or uncertain) accuracy, Sp and Se estimates are. Please define accuracy, Sp and Se for a less specialized audience.

**In this study, leave-one-out cross validation was used to maximize the training set since our datasets were relatively small. 10-fold validations will be used on future, larger datasets to minimize overfitting. Definitions of accuracy, Sp, and Se are now fully described in Methods section.**

- To complement the presented accuracy measures of the model predictions, the ROC curves should be also presented (in the main text) and the respective area under the curve calculated.

- **To our knowledge, it is not possible to calculate ROC curves for classification problems where there is more than one predictor variable.**

- I like the idea of having cutoffs in the covariates/features. This reminds what malaria epidemiologist do in serological data analysis where a cutoff is used to define seronegative and seropositive population. From a perspective of responsible and explicable machine learning, I recommend to fit finite mixture models (Gaussian or non-Gaussian) to the antigen data, check whether there are multiple latent populations (e.g, antigen-negative plus multiple antigen-positive levels), and whether the cutoffs derived from conditional inference trees are related to the discrimination between these latent populations. Particularly flexible finite mixture models are the ones based on the Skew-normal and Skew-t distributions as described in Domingues et al (doi: 10.1101/2021.03.08.21252807). This additional analysis takes the paper into a whole new level.

- **We appreciate this suggestion from the reviewer and will refer to the added comment above which the authors have updated the Discussion section to explicitly mention alternative statistical approaches. Using and/or comparing alternative approaches is outside the scope of this work. Specifically, this current unit of work attempts to move away from more simple antigen positive/negative approaches and maintain antigen concentrations as continuous.**

- In Table 1, accuracy for the infection level data should be discriminated per infection level. I bet misclassification comes mostly from categories related to low parasitemia infections.

- **We thank the reviewer for the suggestion here, but due to relatively smaller datasets utilized here, these accuracy estimates are unable to be reliably generated when sub-dividing the dataset by the 5 infection levels. We have added in Discussion the need to take these (and alternative) approaches on larger datasets.**

- In Table 1, it is interesting that Sp seems to be lower in Angola than in Haiti. I bet this is related to a higher transmission in Angola than in Haiti. This is an interesting finding that deserves exploration and discussion.

- **The authors agree with the reviewer, and have revised the last sentence in the limitations paragraph to point this out directly: “High-transmission areas (like Angola) might not perform as well using this model compared to low-transmission areas (like Haiti) due to lingering HRP2 antigen in circulation (5, 7), which could negatively impact specificity estimates.”**

- In the text, it says the accuracy for the infection levels ranged from 59% and 72%, but the estimates in Table 1 do not show any estimate equal to 72%. Hopefully, this is just a typo.

- **Yes, this was a typo, and has been corrected.**

- I am confused that “As the Angola TES 185 only enrolled participants based on a microscopically-confirmed parasite density above 2,000 p/μL, those data were not able to be evaluated in this categorization scheme” (lines 184—186). It seems this dataset was not used at all for prediction given that it could not also be used for infection status prediction. If that is the case, the paper needs to be totally revised to remove any reference to this dataset (including Figures and Supplementary Figures).

- **Because the Angola microscopy dataset only contained samples >2,000, that was automatically in our “high” infection level status compared to the other Angola (sen-PCR) and Haiti datasets. As the Angola (microscopy) dataset used a different category, those results were only include in the Supplemental. The authors have edited the figure legend to reflect the correct category levels and the text referred to in this comment. The authors have now included the accuracy for this dataset in the main Table 1.**

- What was the rationale to include a principal component analysis (PCA) as it is not use to predict infection status and levels? Given that the objective is related to a classification problem, why not to use a related multivariate technique such as linear discriminant analysis as suggested above?

- **Please see comment above – the authors have updated the discussion to explicitly mention alternative statistical approaches, including linear discriminant analysis. For this current study, PCA was utilized to visualize sub-populations of infection status (as determined by PCR data) as a factor of antigen concentrations.**

- With respect to PCA, I cannot observe that higher values of PC1 reflect high infection levels for Figure 4D (lines 224-225).

- **The authors apologize for the poor resolution of the originally submitted figures, and we have uploaded new figures with higher resolution. The reviewer is correct in that lower PC1 values correspond to higher PCR-determined parasite densities in Figure 4D, and have revised Results text to reflect this.**

- Figures: unfortunately, I could not make a better assessment of the figures due to their low resolution. However, I think violin plots or related plots provide a more informative way of visualizing the data instead of the boxplots shown in Figure 1. In the same Figure, it should be clearly stated in the figure legend that non-infected individuals are not represented in the plots. The remaining Figures are unreadable. What is it plotted in the y axis of the plots at the bottom of the trees? All figure legends should be expanded to be more informative.

- **The authors apologize for the poor figure resolution, and have resubmitted with higher resolution figures. The authors appreciate the suggestion for violin plots, but prefer to stick with boxplots as they accurately depict the data and are more intuitive for the reader. The y-axis for the trees has been added to the figure legends.**

- Code and data sharing: to increase replication of the study by other researchers, authors should consider to share their data and code with the community.

- **The authors thank the reviewer for this comment, and have added all code to the Github, and indicated this at the end of Methods.**
