# Supplementary material for: Predicting Plasmodium falciparum infection status in blood using a multiplexed bead-based antigen detection assay and machine learning approaches
Source: PLoS One. 2022 Sep 29;17(9):e0275096. doi: 10.1371/journal.pone.0275096 (PMC9521833; doi:10.1371/journal.pone.0275096)
Supplement: Attachment — Submitted filename: Response to Reviewers_R1.docx [file pone.0275096.s008.docx]

**Schmedes, et al.**

**PONE-D-21-34503R1**

**Response to Reviewers**

**All references have been cross-checked for accuracy.**

1. In lines 339-346, it should be written more clearly that optimal accuracy might not have been achieved in this study, because this study did not attempt to do it so. To achieve optimal accuracy, other statistical and machine learning methods should have been used and compared with the conditional inference trees.

**- The authors are in agreement with the reviewer’s assessment, and as not every possible method was tested here (with the same dataset), we could not make a statement that the greatest accuracy was achieved. We have revised this section on Line 343 to include the statement: “As such, it could not be stated that this approach utilized here would produce optimal accuracy, and further investigation…”**

2. With respect to my point about cutoffs, I agree that we should aim for using more advanced analytical techniques. However, given that malaria (sero-)epidemiologists are well aware of cutoff-based methods, the paper benefits of explicitly suggesting the link between the cutoffs suggested by the conditional inference trees and those that can be derived from finite mixture models. This suggestion connects the study with the existing literature. This connection can be done by revising the paragraph in lines 339-346.

**- The authors thank the reviewer for this suggestion, but do not agree with the parallels being drawn between the antigen detection data being presented in this current study and antibody detection data (what is utilized for malaria seroepidemiology). Statistical methods for dichotomization, including FMM, have been widely discussed in the antibody (almost unanimously IgG) literature, but this does not apply to levels of *Plasmodium* antigens in blood samples – a completely different biological phenomenon. Our group has casually investigated cutoffs for antigen data in previous studies (cited here), but there is no real precedent in the literature outside of our previous work.**

3. In the Material & Methods, it is important to state that it was assumed that there was no sample contamination in the data analysed.

**- The authors agree with this statement, and have added text to Line 134: “For all laboratory data collected for analyses, it was assumed there was no sample contamination.”**

4. Sorry for raising this comment at this stage, but it seems that dataset is imbalanced and this might affect the accuracy predicted by conditional inference trees. This should be briefly discussed by acknowledging that there are (machine learning) methods that could have been used to correct for that.

**- For analyses presented here, datasets from separate studies were analyzed independent of one another, and predicted accuracies were presented for each unique study/analysis. So while the authors would agree that categories are not equally represented within each dataset, we provide datasets from different malaria transmission settings as examples of how these outputs may be interpreted from different endemic settings. The authors feel they currently describe the limitations of the current datasets in Discussion, but we have now added additional text to explicitly state that ML methods exist to help deal with imbalanced datasets on Line 340: “Future studies on larger datasets should address optimal statistical tests and machine learning models for infection status prediction, as well as employ methods to correct for dataset imbalance.”**
